# First report of L1014F-*kdr* mutation in *Culex pipiens* complex from Morocco

**DOI:** 10.1186/s13071-016-1931-5

**Published:** 2016-12-16

**Authors:** Meriem Bkhache, Fatim-Zohra Tmimi, Omar Charafeddine, Chafika Faraj, Anna-Bella Failloux, M’hammed Sarih

**Affiliations:** 1 Institut Pasteur du Maroc, Laboratoire des Maladies Vectorielles, Place Louis Pasteur, Casablanca, 20360 Morocco; 2 Faculté des Sciences et Techniques de Mohammedia, Laboratoire de Virologie Microbiologie & Qualité/Eco-toxicologie & Biodiversité, Université Hassan II de Casablanca, Casablanca, Morocco; 3 Institut National d’Hygiène, Laboratoire d’Entomologie Médicale, Rabat, Morocco; 4 Institut Pasteur, Department of Virology, Arboviruses and Insect Vectors, 25-28 rue du Docteur Roux, Paris, 75724 France

**Keywords:** *Culex pipiens*, L1014F *kdr*, Lambda-cyhalothrin, Resistance, Morocco

## Abstract

**Background:**

Mosquitoes of the *Culex pipiens* complex, competent vectors for West Nile virus (WNV) and Rift Valley fever virus (RVFV) are widely targeted by insecticide treatments. The intensive application of chemical insecticides led to the development of resistance in many insects including *Culex pipiens* mosquitoes. The absence of data on resistance mechanisms in Morocco allow us to assess the levels of lambda-cyhalothrin resistance and the frequency of the mutated gene L1014F *kdr* in different forms of *Cx. pipiens* complex from three regions of Morocco.

**Methods:**

Mosquito adults were reared from immature stages collected in three different regions in Morocco (Tangier, Casablanca and Marrakech). Standard WHO insecticide susceptibility tests were conducted on adults emerged from collected larvae. Specimens were identified as belonging to the *Culex pipiens* complex using a multiplex PCR assay with diagnostic primers designed from the flanking region of microsatellite CQ11. Identified mosquitoes were then tested for the presence of the L1014F *kdr* mutation using PCR assay.

**Results:**

Our results showed that 21% of the tested population has a resistance to lambda-cyhalothrin. The molecular identification of survivors shows that 43% belonged to the *Cx. pipiens pipiens* and only 9.5% to the *Cx. pipiens molestus* form. On the other hand, 416 specimens were screened for the L1014F *kdr* mutation. L1014F mutation was detected in different forms of *Cx. pipiens* in different sites. The frequency of L1014F mutation was similar between the *Cx. pipiens pipiens* form and hybrid form, while it was lower in the *Cx. pipiens molestus* form. The presence of the L1014F *kdr* allele was significantly associated with resistance to lambda-cyhalothrin in *Cx. pipiens pipiens* (*P* < 0.0001) and hybrid form (*P* < 0.0001).

**Conclusion:**

Resistance to lambda-cyhalothrin of *Cx. pipiens* populations appears to be largely due to the L1014F *kdr* mutation. To our knowledge, the frequencies of L1014F *kdr* mutation are examined for the first time in natural populations of the *Culex pipiens* complex in Morocco. These findings will provide important information to propose more adapted vector control measures towards this mosquito species, potential vector of arboviruses.

## Background

Mosquitoes of the *Culex pipiens* complex are potential vectors of Rift Valley fever virus (RVFV) and West Nile virus (WNV). RVFV is a *Phlebovirus* of the family *Bunyaviridae*, considered as an emerging zoonotic vector-borne disease representing a threat to animal and human health, and livestock production mainly in sub-Saharan Africa [1]. It causes abortions and high mortalities in newborn animals [2, 3], and in humans, it gives different symptoms varying from a flu-like syndrome to hemorrhagic manifestations with a case fatality rates as high as 50% [4]. Besides, WNV is an arbovirus of the family *Flaviviridae* and the genus *Flavivirus*. It has an extensive distribution throughout Africa, the Middle East, southern Europe, western Russia, south-western Asia and Australia.

Widely spread in North Africa, *Culex pipiens* complex is a competent vector of several pathogens affecting human and/or animals such as WNV [5] and RVFV [6]. In Morocco, *Culex pipiens* mosquitoes have been strongly suspected as being the vectors of WNV during epizootics in 1996 with 42 dead horses [7–9] and in 2003 [10]. In the Maghreb region, WNV was repeatedly responsible for several outbreaks: Algeria (1994), Tunisia (1997, 2003, 2010–2012), and Morocco (1996, 2003 and 2010) [10–12].

*Culex pipiens* includes two forms, *pipiens* and *molestus*, which are morphologically identical but genetically different. They are also distinguishable by their physiological and behavior differences. *Pipiens* form is anautogenous (needs a blood meal for eggs development), ornithophilic (feeds on birds), heterodynamic (enters into diapause in winter), and eurygamous (prefers mating in large and open spaces), whereas *molestus* form is autogenous (lays the first egg batches without feeding on blood), mammophylic (feeds on mammals), homodynamic (is active throughout the year), and stenogamous (mates in closed areas) [13]. In the absence of effective vaccines, the control of mosquito populations remains the unique measure to limit pathogen transmission. Thus, the use of insecticides plays a major role in the prevention and control of vector-borne diseases. However, the frequent use of insecticides (mainly pyrethroids and organophosphates) has contributed to select several resistance mechanisms in targeted mosquito populations. There are two mechanisms of resistance: (i) increased production of detoxifying enzymes such as cytochrome P450 oxidases or glutathione-S-transferases; and (ii) modification of insecticides targets as the synaptic acetylcholinesterase (AchE1) encoded by ace-1 gene, the γ-aminobutyric acid (GABA) receptor gene encoded by *Rdl* and the voltage-dependant sodium channel encoded by *kdr* [14, 15]. Pyrethroids (PYR) target Sodium channels; this neurotoxin insecticide binds to the Na + channel and then prolongs depolarization [16–18]. The magnitude of the PYR effect depends on the type of insecticide molecule: the type I insecticide (e.g. permethrin) does not present a cyano group compared to the type II insecticide (e.g. lambda-cyalothrin and delmathrin). The type II insecticides induce a more acute effect as they produce longer depolarization [17]. Phenotypically, Na + channels inactivation results in a rapid knockdown (*kd*) of mosquitoes leading in some cases to death. Resistance to *kd* is caused by a mutation L1014F, the substitution of a leucine at position 1,014 by a phenylalanine conferring the *kdr* phenotype [19], leading to a lower sensitivity of receptors to these insecticides and modifying the potential action of the channel [18, 20].

In Morocco, the mechanisms responsible for insecticide resistance in *Cx. pipiens* remain unknown; the only data available describes the level of larval resistance to the OP insecticide temephos [21]. Knowing that insecticide resistance remains a global issue for the control of mosquito-borne diseases, this study aims to investigate the L1014F *kdr* mutation frequencies in different forms of *Culex pipiens* complex collected in three regions in Morocco: Tangier, Casablanca and Marrakech.

## Methods

### Collection sites

**Fig 1:**
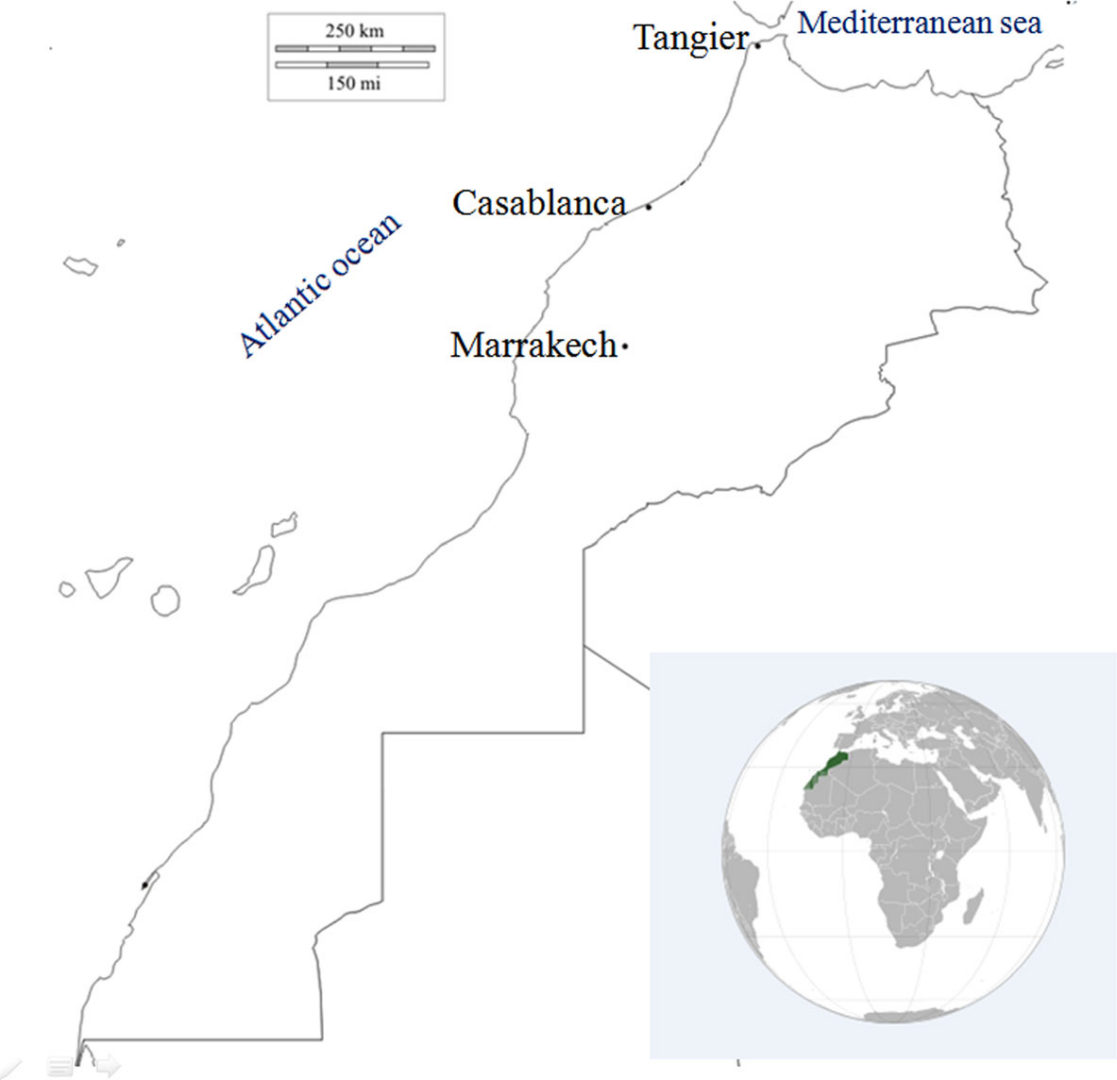
Localization of *Culex pipiens* collection sites in Morocco

Mosquitoes were collected as larvae using the “dipping” sampling method during summer 2015 from three Moroccan regions (Fig. 1). Sampling was carried out in three bioclimatic zones: humid (Tangier), semi-arid (Casablanca) and arid (Marrakech). In each region, we have selected two sites: an urban site (in the center of the city) and a rural site (either in villages or in the city outskirts where inhabitants live at close proximity with planted areas and domestic animals). Fourth instar larvae were used for morphological identification and reared until imago stage at 28 ± 1 °C with 80% relative humidity and a 16 h:8 h photoperiod. Mosquitoes were identified as *Culex pipiens* using a dichotomous key for the identification of the Culicidae in the Mediterranean area [22].

### Insecticide susceptibility test

Adult bioassays were conducted using four batches of 20–25 females. One-three day-old unfed females were exposed for 1 h to insecticide-impregnated 0.05% lambda-cyhalothrin according to World Health Organization (WHO) recommendations. As a control, 50 non-blood-fed females mosquitoes were exposed to insecticide-free papers. The number of mosquitoes knocked down while were exposed to insecticide was recorded at intervals of 10 min, and then the percentage of mortality was calculated at 24 h post-exposure. Dead and surviving mosquitoes were conserved at -20 °C for molecular species identification and *kdr* analysis.

### Identification of *Culex pipiens* forms

DNA was extracted individually from mosquitoes using the method of DNAzol as described in the manufacturer’s protocol. Specimens were identified as *Culex pipiens* complex using a multiplex PCR assay described in Bahnck & Fonseca [23]. The locus CQ11 was used to distinguish between the forms of *Cx. pipiens*: *pipiens*, *molestus* and hybrid.

### Detection of *Kdr* mutation

For the detection of *kdr* mutation, two separate PCRs were run, one to detect alleles of the leucine-phenylalanine substitution and the other to detect wild-type susceptible alleles following the methods described in Martinez-Torres et al. [24]. DNA fragments were separated by electrophoresis on 1.5% agarose gel with ethidium bromide and viewed under ultraviolet light.

The genotype frequencies were calculated by dividing the number of individuals with a given genotype by the total number of analyzed mosquitoes as follows: (i) homozygous wild type genotype frequency L1014/L1014, (ii) homozygous mutant genotype frequency, F1014/F1014, and (iii) heterozygote genotype frequency, L1014/F1014.

### Data analysis

A categorical variable was compared by Fisher’s exact test and Chi-square test. The association between the L1014F *kdr* genotype frequencies and lambda-cyhalothrin resistance phenotypes was estimated by the odds ratio (OR) and its corresponding 95% confidence interval (CI). Differences between groups were considered significant for *P*-values less than 0.05. All tests were two sided.

## Results

### Insecticide susceptibility and identification of *Culex pipiens* forms

Twenty-four hours after exposure of 100 *Cx. pipiens* collected in Casablanca to lambda-cyhalothrin, 79% of exposed adults died. KDT50 and KDT90 were 27 min and 42 min, respectively.

Insecticide-resistant and insecticide-susceptible adults after insecticide bioassays were tested by PCR to identify the *Culex pipiens* form. Most *Cx. pipiens* resistant to lambda-cyhalothrin were *pipiens* (43%, 9/21) and hybrids (47.5%, 10/21) while *molestus* represented only 9.5% (2/21). Besides, *Cx. pipiens* susceptible to lambda-cyhalothrin were mainly *pipiens* (36.5%, 29/79) and hybrids (36.5%, 29/79) while 27% (21/79) were *molestus*.

### *Kdr* gene detection

One hundred *Cx. pipiens* adults collected in Casablanca were tested for the *kdr* mutation. The frequency of genotypes was represented in Table 1.

**Table 1:** Frequencies of *kdr* mutation according to the phenotypic status (resistant/susceptible) of different forms of *Cx. pipiens* in Casablanca

				Genotype (%)		
Forms of *Cx. pipiens*	Phenotype	*N* (%)	1014 L/1014 L *n* (%)	1014 L/1014 F *n* (%)	1014 F/1014 F *n* (%)	Frequency of allele 1014 F (%)
*Cx. pipiens pipiens*	Susceptible	29 (76)	29 (100)	0	0	0
	Resistant	9 (24)	3 (33)	5 (56)	1 (11)	0.39
Hybrid	Susceptible	29 (74)	29 (100)	0	0	0
	Resistant	10 (26)	2 (20)	4 (40)	4 (40)	0.6
*Cx. pipiens molestus*	Sucseptible	21 (91)	21 (100)	0	0	0
	Resistant	2 (9)	2 (100)	0	0	0

*Abbreviation*: *N*, number of individuals tested

All specimens of susceptible mosquitoes had 1014 L/1014 L genotype. Among the 21 resistant mosquitoes, 9 had 1014 F/1014 F genotype, 5 had 1014 F/1014 L genotype and 7 had 1014 L/1014 L genotype (Table 2). *Culex pipiens pipiens* and hybrids showed a significant correlation between the *kdr* resistant allele 1014 F and the resistant phenotype to lambda-cyhalothrin with OR = 76.3 (*P* < 0.0001) and OR = 172.1 (*P* < 0.0001), respectively (Table 2).

**Table 2:** Correlation between the frequency of 1014 F allele and insecticide-resistance/-susceptible phenotypes to lambdacyalothrin

Form of *Cx. pipiens*	Phenotype	*N*	Alleles		Odds ratio	*P*-value
			1014 F (R)	1014 L (S)		
*Cx. pipiens pipiens*	Resistant	9	7	11	76.3	0.0001
	Susceptible	29	0	58	4.06–1432	
	Total	38	9.2%	90.8%		
Hybrid	Resistant	10	12	8	172.1	0.0001
	Susceptible	29	0	58	9.3–3182	
	Total	39	14.4%	85.6%		
*Cx. pipiens molestus*	Resistant	2	0	4	9.0	1
	Susceptible	21	0	42		
	Total	23	0%	100%		

*Abbreviation*: *N*, number of individuals tested

### Frequencies of *Culex pipiens* forms in three sites

A total of 452 adults collected in Tangier, Casablanca and Marrakech were characterized by PCR and frequencies of different forms of *Cx. pipiens* are presented in Table 3. *Culex pipiens pipiens* and *Cx. pipiens molestus* and also their hybrids were found in urban and rural habitats. 49.9% of tested mosquitoes were *pipiens* form; 32.3% were hybrid and 20.8% were *molestus* form (Table 3).

**Table 3:** Numbers and frequencies of *Culex pipiens* forms in Morocco (Tangier, Casablanca, Marrakech). *Culex pipiens* larvae were collected at different sites in Morocco, reared to adults and identified by PCR amplification of the flanking region of the CQ11 microsatellite. Frequencies of tested mosquitoes are in parentheses

	Tangier			Casablanca			Marrakech		
	Rural	Urban	Total	Rural	Urban	Total	Rural	Urban	Total
	*n* (%)	*n* (%)	*n* (%)	*n* (%)	*n* (%)	*n* (%)	*n* (%)	*n* (%)	*n* (%)
*Cx. pipiens pipiens*	48 (55)	45 (66)	93 (60)	38 (25)	35 (83)	73 (37)	20 (35)	26 (59)	46 (45.5)
Hybrid	37 (43)	22 (32)	59 (38)	36 (23)	5 (12)	41 (21)	28 (49)	18 (41)	46 (45.5)
*Cx. pipiens molestus*	2 (2)	1 (2)	3 (2)	80 (52)	2 (5)	82 (42)	9 (16)	0 (0)	9 (9)
Total	87	68	155	154	42	196	57	44	101

### Frequencies and distribution of 1014 F allele in three sites

A total of 416 *Cx. pipiens* samples were examined. In Tangier, 143 individuals were tested for the 1014 F *kdr* mutation: 185 samples in Casablanca, and 88 in Marrakech. The *kdr* mutation was detected in the different forms of *Cx. pipiens* in different sites of three cities in Morocco. The frequency of the 1014 F *kdr* allele was similar between *pipiens* form and the hybrid form (*χ* ^2^ = 1.02, *df* = 1, *P* = 0.312) while there was a significant difference of frequencies between *pipiens* form and *molestus* form (*χ* ^2^ = 57.11, *df* = 1, *P* < 0.0001) and between *molestus* form and hybrid form (*χ* ^2^ = 44.23, *df* = 1, *P* < 0.0001). The frequencies were not significantly different between Tangier and Marrakech (*χ* ^2^ = 2.33, *df* = 1, *P* = 0.127) (Table 4).

**Table 4:** Frequencies of the 1014 F *kdr* allele

City	Site	*Cx. pipiens pipiens*			Hybrid			*Cx. pipiens molestus*		
		*N*	%	95% CI	*N*	%	95% CI	*N*	%	95% CI
Tangier	Rural	45	0.44	0.34–0.54	36	0.39	0.28–0.50	2	1	1
	Urban	38	0.24	0.14–0.34	21	0.38	0.23–0.53	1	0	0
	Total	83	0.35	0.28–0.42	57	0.38	0.29–0.47	3	0.67	0.29–1.05
Casablanca	Rural	38	0.17	0.08–0.09	36	0.25	0.15–0.35	80	0	0
	Urban	25	0.32	0.19–0.45	4	0.25	0.00–0.25	2	0.25	0.00–0.67
	Total	63	0.23	0.16–0.30	40	0.35	0.25–0.45	82	0.006	0.00–0.018
Marrakech	Rural	20	0.35	0.20–0.47	28	0.21	0.10–0.32	9	0.33	0.11–0.55
	Urban	18	0.25	0.11–0.39	13	0.46	0.27–0.65	0	0	0
	Total	38	0.30	0.20–0.40	41	0.29	0.19–0.39	9	0.33	0.11–0.55
	Total	184	0.3	0.25–0.34	138	0.32	0.26–0.37	94	0.095	0.05–0.14

*Abbreviations*: *N* number of individuals tested, *CI* confidence interval

## Discussion

To the best of our knowledge, we report for the first time in Morocco the resistance status of different forms of *Cx. pipiens* and also the frequency of the L1014F *kdr* mutation in field populations. We found that *Cx. pipiens pipiens* was more resistant than *Cx. pipiens molestus*: 43 and 9.5%, respectively. We also found that *Cx. pipiens pipiens* and *Cx. pipiens molestus* and their hybrids, co-occur in aboveground and underground breeding sites in urban, and rural habitats.

In Morocco, vector control programs use pyrethroids to treat adults as this insecticide family presents a high efficacy and low human toxicity [25, 26]. However, we showed that these insecticide treatments were correlated with high frequencies of 1014 F/1014 L genotype in field-collected mosquitoes. The L1014F *kdr* mutation which affects the voltage gated sodium channel gene is one of the mechanisms of resistance against dichlorodiphenyltrichloroethane (DDT) and pyrethroids group of insecticides. Culex pipiens mosquitoes present a high resistance to pyrethroids, organophosphates and carbamates in many regions of Morocco with variable levels according to regions (data not published). Unexpectedly, we found that some mosquitoes presenting a resistant phenotype were homozygous for the kdr susceptible allele 1014 L. This surprising result previously described by other teams [27] underlines that other resistance mechanisms can be involved. The presence or absence of *kdr* mutation gives no indicationof the actual strength of resistance level. The presence of *kdr* mutation alone cannot inform of the operational impact of the resistance. Even if the kdr mutation ispresent, contribution of other resistance mechanisms such as metabolic resistance could also play a crucial role in the impact of resistance. Resistance toinsecticides is an evolutionary phenomenon. The factors which condition its evolution depend at the same time on the biology of the insect, on the nature of the mechanisms involved and on the operational aspects of treatments. The study of the evolution resistance genes in vector populations is very important. It allows to assess the impact of the resistance on the efficacy of the vector control. In fact, the operational implications of resistance are not directly deductible only from the level of resistance measured in the laboratory. Even if the resistance is present, it might not yet have an operational impact and this is why monitoring the resistance intensity in the population is important.

A total of 416 specimens were investigated for L1014F *kdr* mutation. The L1014F mutation remains widespread in all three ecological regions. A higher proportion of heterozygous 1014 L/1014 F genotype for *kdr* mutation was found in Tangier and Marrakech, 66 and 60%, respectively. It is known that the frequencies of *kdr* heterozygous 1014 L/1014 F genotypes were highly variable ranging from 14 to 80% depending on location sites [28–30]. Widespread use of pyrethroids within households may explain the high frequency of the *kdr* mutation in urban areas. Moreover, the extensive use of pesticides in agriculture could also contribute to select the *kdr* mutation in mosquito populations. Unlike the heterozygous 1014 L/1014 F genotypes, we found that the frequency of homozygous 1014 F/1014 F genotype was very low. The low proportion of homozygous 1014 F/1014 F *Cx. pipiens* can be consistent with a high fitness cost associated with the *kdr* mutation. Additional studies are required to explore this hypothesis. The L1014F mutation has been reported at least in 39 arthropod species of which six are mosquitoes, three *Culex* spp. and three *Anopheles* spp. It has been reported in *Cx. pipiens* mosquitoes in at least 14 countries [31, 32]. It has been shown that the L1014F provides variable levels of protection to Type I or Type II pyrethroids [33]. The extensive use of pyrethroids for personal protection in urban environments, the recently introduced Ultra Low Volume (ULV) sprays against mosquitoes, as well as the long-term use of pyrethroids may have accelerated the selection of pyrethroid resistance mutations [34]. It has been shown that the L1014F mutation conferred a resistance to permethrin (Type I) in *Cx. p. quinquefasciatus* [35, 36] and to deltamethrin (Type II) in *Cx. pipiens pallens* [37].

## Conclusions

Our work showed that *Cx. pipiens* was resistant to lambda-cyhalothrin 0.05% and that the *pipiens* form was more resistant than the *molestus* form. Also, we described for the first time the distribution and the frequency of *kdr* mutation in *Cx. pipiens* complex from Morocco. These data provide suitable information for the design and implementation of successful resistance management strategies against this species, potential vector of arboviruses and to establish reliable diagnosis methods. Detection of specific pyrethroid resistance mutation can help to track and map the spread of resistance and also to assess the response of mosquito populations to future insecticide-based interventions.

## Abbreviations

AChE1: Acetylcholinesterase-1 enzyme
CI: Confidence interval
*Cx*: *Culex*
DDT: Dichlorodiphenyltrichloroethane
GABA: Gamma-aminobutyric acid
Kd: Knockdown
*Kdr*: Knockdown resistance
OP: Organophosphates
OR: Odds ratio
PCR: Polymerase chain reaction
PYR: Pyrethroids
RVFV: Rift valley fever virus
ULV: Ultra-low volume
WHO: World Health Organization
WNV: West Nile virus
